# Knowledge, Attitude, and Practice of Lifestyle Modifications Among Saudi Women Diagnosed With Polycystic Ovary Syndrome (PCOS)

**DOI:** 10.7759/cureus.49398

**Published:** 2023-11-25

**Authors:** Amal Aljuaid, Hala A Sindi, Wajd Alhadi, Ishtiyaq A Abu Zayied, Lama Althobaiti, Iffat Imran

**Affiliations:** 1 Medicine, Taif University, Taif, SAU; 2 Medicine and Surgery, Faculty of Medicine, King Abdulaziz University, Jeddah, SAU; 3 Medicine, King Khalid University, Abha, SAU; 4 Medicine, University of Tabuk, Tabuk, SAU; 5 Obstetrics and Gynaecology, Taif University, Taif, SAU

**Keywords:** physical activity, quality of life, diet modification, saudi women, health education & awareness, polycystic ovary syndrome (pcos)

## Abstract

Background

Polycystic ovary syndrome (PCOS) is a common endocrine disorder in women, often associated with metabolic issues like obesity and insulin resistance. Lifestyle changes, including weight loss, healthy eating, and regular exercise, are recommended for PCOS management. Studies have explored women's perspectives on these changes, revealing misconceptions and adherence challenges. Recognizing the importance of individualized interventions, particularly addressing knowledge gaps, is vital for improving the quality of life for women with PCOS, especially in cultural contexts like Saudi Arabia.

Methodology

This was a cross-sectional study conducted in Saudi Arabia from May to August 2023 including PCOS patients. Data was collected through questionnaires and analyzed using IBM SPSS 29 (IBM Corp. Released 2020. IBM SPSS Statistics for Windows, Version 27.0. Armonk, NY: IBM Corp). This study was conducted in accordance with the ethical guidelines and principles outlined by the Scientific Research Ethics Committee of Taif University (no.44-359). All participants provided informed consent, and the study protocols, including data collection, analysis, and publication, adhered to the relevant ethical standards.

Results

Our study included a majority aged 18-29 (27.4%), married individuals (55.6%) and those with a Bachelor's degree (72%). About 46.9% were medically diagnosed with PCOS. A notable proportion (70.2%) reported no family history of PCOS. Doctors were the primary information source (40.7%). Knowledge about PCOS was generally high, with correct recognition of various PCOS characteristics and treatment options. Attitudes were positive, especially among medically diagnosed individuals, and 91% believed weight reduction could improve PCOS symptoms. Lifestyle modification knowledge, attitude, and practices showed significant associations with demographic factors like age, place of residence, education, marital status, working in healthcare sectors, and PCOS diagnosis status.

Conclusions

Knowledge about PCOS is generally high among women with positive attitudes toward its management through lifestyle modifications. Women generally show positive practices of lifestyle modifications in PCOS, and they are associated with sociodemographic features.

## Introduction

Polycystic ovarian syndrome (PCOS) is a condition caused by hormonal imbalance in women of reproductive age [1]. The symptoms include ovulating problems, hyperandrogenism, and ovarian cysts [2]. PCOS is associated with metabolic problems such as obesity, insulin resistance, and hypercholesterolemia, which might lead to severe complications like type two diabetes and cardiovascular diseases [3]. However, a healthy lifestyle, such as a balanced diet, regular exercise, and weight loss, has been shown to improve metabolic health in women with PCOS and prevent further complications [4,5]. Based on the counsel of experts such as the American Association of Clinical Endocrinologists, the American College of Endocrinology, and the Androgen Excess and PCOS Society Disease State Clinical Review [6], the initial treatment of PCOS should begin with lifestyle modification. They also stated that women with PCOS are recommended to lose 5%-10% of their body weight, follow a healthy diet, and exercise regularly [7]. These changes showed to benefit PCOS-affected women in terms of their health and infertility outcomes. Various researchers have studied the knowledge, attitudes, and practices of women with PCOS regarding lifestyle changes. For example, in Taif, Saudi Arabia, Albezrah and Arein (2019) studied women’s perspectives on weight loss, which has revealed misconceptions about the condition and a lack of consistency in adopting healthy dietary and exercise habits [8]. Furthermore, a study conducted in Australia by Ranasinha et al. (2015) found that Australian women with PCOS have a high prevalence of metabolic risk factors, which healthy lifestyle choices can reduce [9]. Moreover, a study by Cowan et al. (2023) pointed out that lifestyle adjustment may lead to improved metabolic disorders like insulin resistance, glucose tolerance, and lipid profiles among women with PCOS [5]. These findings emphasize the significance of individualized interventions considering cultural, social, and psychological factors when managing PCOS. To sum it up, the effectiveness of lifestyle modification in managing PCOS in women has been observed. In our research, we will measure the extent of the gaps in knowledge of Saudi women with polycystic ovary syndrome to make it easier for doctors to address these misconceptions and improve the quality of life of the patients.

## Materials and methods

Study design and subjects

The study employed a cross-sectional design. Data was collected by a randomized, self-administered questionnaire, which was sent to the respondents electronically via e-mail from May 25, 2023, to August 25, 2023. The research was carried out in a questionnaire-based setting and comprised four sections: A) Sociodemographic data will be collected such as age, residency, education level, nationality, place of residence, and marital status. B) Knowledge of participants about polycystic ovarian syndrome such as did you heard about PCOS before, source of knowledge, what are the problems of PCOS, and methods of treatment. C) Attitude and practice of the PCOS patients about weight reduction and/or lifestyle modification such as weight reduction is an effective treatment if yes how does weight reduction improve your condition or if no why not effective? D) Daily practices of the PCOS patients such as low low-fat foods, eating smaller portions at dinner, and exercising for 30 minutes. To establish the sample size, the Raosoft sample size calculator (Raosoft Inc., Seattle, WA, USA, raosoft.com) was utilized, resulting in a requirement for 385 PCOS (polycystic ovary syndrome) patients to achieve a 95% confidence interval with a 5% margin of error. The study included all registered PCOS patients residing in Saudi Arabia, excluding individuals who did not suffer from PCOS.

Data analysis 

Data was collected using an Excel (Microsoft Corporation, 2018) sheet for coding, and subsequent analysis was conducted with IBM SPSS version 27 (IBM Corp. Released 2020. IBM SPSS Statistics for Windows, Version 27.0. Armonk, NY: IBM Corp). For continuous variables, measures of central tendency were computed, while frequencies and percentages were estimated for categorical variables. In the case of continuous variables, the comparison was performed using the Wilcoxon Mann-Whitney test, represented as mean ±. Categorical variables were analyzed using independent sample t-tests, analysis of variance, and the Chi-Square test of independence, with results expressed in absolute values and percentages.

Ethical considerations 

This study was conducted in accordance with the ethical guidelines and principles outlined by the Scientific Research Ethics Committee of Taif University (no. 44-359). All participants provided informed consent, and the study protocols, including data collection, analysis, and publication, adhered to the relevant ethical standards.

## Results

Our study included 379 participants. The majority were aged 18-29 (N=104, 27.4%), married (N=211, 55.6%), and had a Bachelor's degree (N=273, 72.0%). A significant proportion had a normal BMI (N=226, 59.6%), were Saudi (N=356, 93.9%), and were not healthcare workers (N=299, 78.9%). Most resided in the western region (N=226, 59.6%) and had a monthly income >10,000 SAR (N=183, 48.3%). About 46.9% (N=178) were medically diagnosed with PCOS, while 46.2% (N=176) were not diagnosed, and 6.9% (N=26) were suspected cases. Regarding family history of PCOS, the majority of participants (N=266, 70.2%) reported no family history of PCOS, while a minority (N=113, 29.8%) indicated a positive family history. Among those with a family history, N=12 participants (3.2%) mentioned mothers, and N=20 (5.2%) specifically mentioned a sister as having PCOS (Table 1).

**Table 1:** Sociodemographic of participants PCOS: polycystic ovary syndrome, Hx: history.

		Frequency (N)	Percent (%)
Age	18-24 years	104	27.4
	24-29 years	91	24.0
	30-39 years	63	16.6
	40-45 years	56	14.8
	>46 years	65	17.2
Marital status	Married	211	55.6
	Single	149	39.3
	Divorced/widowed	19	5.0
BMI	18-24.9 kg/m^2^ (normal)	226	59.6
	25-29.9 kg/m^2^ (overweight)	151	39.8
	>30 kg/m^2^ (obese)	2	0.5
Nationality	Non-Saudi	23	6.1
	Saudi	356	93.9
Educational level	Bachelor's degree	273	72.0
	High school degree	78	20.6
	Master's degree	28	7.3
Are you a healthcare worker	No	299	78.9
	Yes	80	21.1
Area of residency	Eastern region	74	19.5
	Northern region	49	12.9
	Southern region	30	7.9
	Western region	226	59.6
Monthly income	<10,000 SAR	123	32.5
	>10,000 SAR	183	48.3
	<25,000 SAR	54	14.2
	25,000-40,000 SAR	17	4.5
	>40,000 SAR	2	0.5
Status of PCOS diagnosis	Medically diagnosed	178	46.9
	Not diagnosed	175	46.2
	Suspected	26	6.9
Diagnosed for how long	Recently (less than 2 years)	90	23.7
	2-5 years	63	16.6
	5-10 years	49	12.9
Family Hx of PCOS	No	266	70.2
	Yes	113	29.8
Relationship	Mom	12	3.2
	Sister	20	5.2

**Figure 1:**
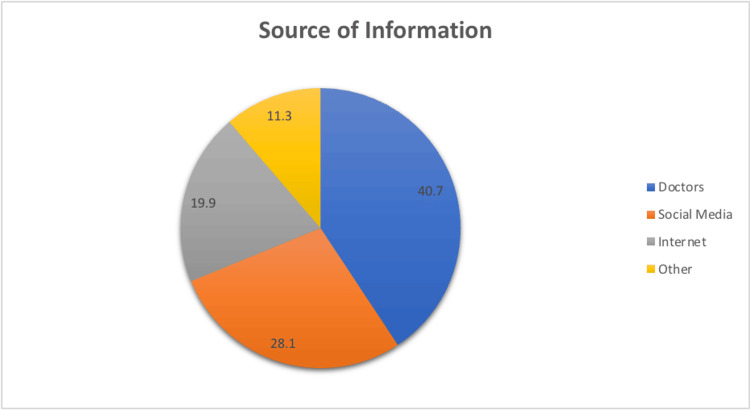
Source of Information about PCOS PCOS: polycystic ovary syndrome.

Regarding the sources of information about PCOS, the most common source was doctors, with 40.7% (N=155) of participants obtaining information from doctors and other advanced practice providers. A smaller percentage relied on social media (N=107, 28.1%), internet (N=72, 18.9%), and other sources (N=43, 11.3%) for their knowledge about PCOS (Figure 1).

Regarding knowledge, the majority had heard about PCOS (N=314, 82.8%) and were aware of androgens' role (N=290, 76.5%) and believed androgens increased in PCOS (N=284, 74.9%). Participants correctly identified characteristics of PCOS, such as small ovarian cysts (N=337, 88.9%), obesity as a potential cause (N=314, 82.8%), pre-diabetes (N=271, 71.5%), and irregular menstrual cycles (N=352, 92.9%). About 85.5% (N=324) recognized unusual hair growth on different body parts as a sign, 68.6% (N=260) associated severe acne during the menstrual cycle, and 75.7% (N=287) identified abnormal/high hair loss from the scalp. Moreover, 84.7% (N=321) knew that PCOS could be confirmed by vaginal ultrasound, and 55.9% (N=212) knew of specific blood tests for diagnosis. Attitudes toward PCOS showed that most recognized its potential links to diabetes (N=174, 45.9%), heart diseases (N=121, 31.9%), infertility (N=342, 90.2%), and anxiety/depression (N=345, 91%). Additionally, participants were generally aware of various treatment options for PCOS, including hormonal therapy (N=310, 81.8%), anti-diabetics (N=246, 64.9%), symptomatic treatments (N=232, 61.2%), and surgery (N=300, 79.2%). Weight reduction was seen as a potential means to improve PCOS symptoms (N=346, 91%) (Table 2).

**Table 2:** Assessment of knowledge and attitude toward PCOS (n=379) PCOS: polycystic ovary syndrome, OCP: oral contraceptive pill.

		Frequency (N)	Percent (%)
Knowledge about PCOS			
Heard about PCOS	No	65	17.2
	Yes	314	82.8
Heard about androgens (testosterone)	No	89	23.5
	Yes	290	76.5
Increased androgens in PCOS	No	95	25.1
	Yes	284	74.9
PCOS patients have small multiple cysts in ovaries	No	42	11.1
	Yes	337	88.9
Obesity causes PCOS	No	65	17.2
	Yes	314	82.8
Pre-diabetes causes PCOS	No	108	28.5
	Yes	271	71.5
Irregular/absence of menstrual cycle is a sign of PCOS	No	27	7.1
	Yes	352	92.9
Unusual hair growth on different body parts	No	55	14.5
	Yes	324	85.5
Severe acne during the menstrual cycle is a sign of PCOS	No	119	31.4
	Yes	260	68.6
Abnormal/high hair loss from the scalp is a symptom of PCOS	No	92	24.3
	Yes	287	75.7
PCOS confirmed by vaginal ultrasound	No	58	15.3
	Yes	321	84.7
Specific blood test can diagnose PCOS	No	167	44.1
	Yes	212	55.9
Attitude toward PCOS			
PCOS may lead to diabetes	No	205	54.1
	Yes	174	45.9
PCOS may lead to heart diseases	No	258	68.1
	Yes	121	31.9
PCOS may lead to infertility/decrease fertility	No	37	9.8
	Yes	342	90.2
PCOS may lead to anxiety/depression	No	34	9.0
	Yes	345	91.0
Hormonal therapy (OCP) may treat PCOS	No	69	18.2
	Yes	310	81.8
Anti-diabetics (metformin) may treat PCOS	No	133	35.1
	Yes	246	64.9
PCOS may be treated symptomatically (clomiphene, letrozole, acne creams, spironolactone)	No	147	38.8
	Yes	232	61.2
Surgery may treat PCOS	No	79	20.8
	Yes	300	79.2
Weight reduction may improve PCOS symptoms	No	34	9.0
	Yes	345	91.0

**Figure 2:**
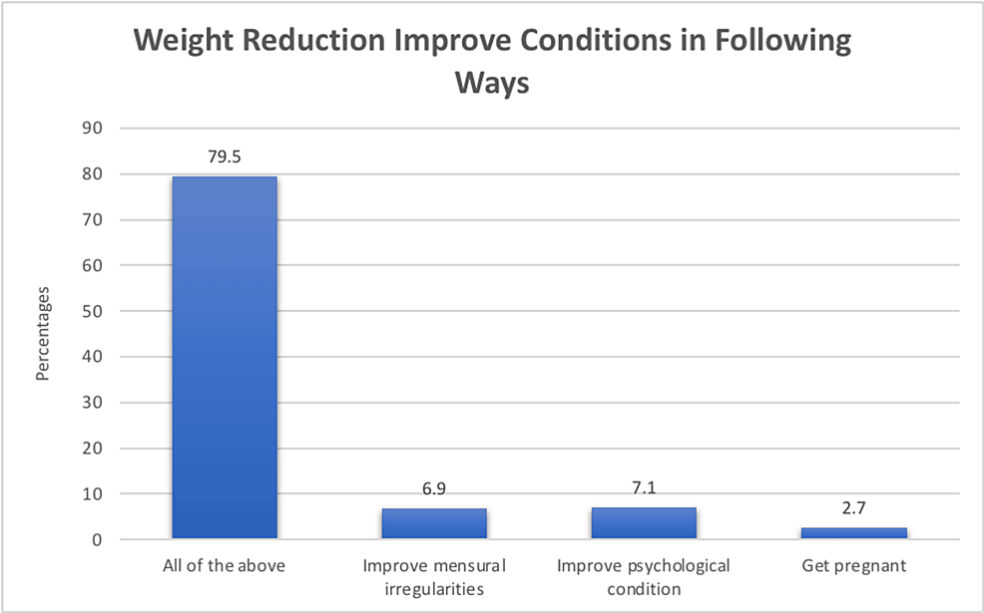
Benefits of weight reduction in PCOS PCOS: polycystic ovary syndrome.

Participants recognized various benefits of weight reduction in managing PCOS. A minority of participants believed it could lead to improvements in multiple aspects, including menstrual irregularities (N=27, 6.9%), psychological condition (N=28, 7.1%), and the possibility of getting pregnant (N=11, 2.7%). A significant portion (N=302, 79.5%) acknowledged that it could have an overall positive impact on PCOS (Figure 2).

Practices include incorporating low-salt foods, eating fruits and vegetables, reducing refined sugar, consuming high-fiber foods, and exercising. Hormonal preparations are used to manage irregular periods, acne, and infertility. Some participants use herbal medications and topical creams. Laparoscopic ovarian drilling is less common (Table 3).

**Table 3:** Assessment of practice regarding lifestyle modifications in PCOS (n=379) PCOS: polycystic ovary syndrome.

		Never	Rarely	Sometimes	Usually	Always
Obesity						
Incorporated low-salt foods in diet	N	61	40	135	82	61
	%	16.1	10.6	35.6	21.6	16.1
Eat 5 servings of fruits and vegetables per day	N	52	53	131	96	47
	%	13.7	14.0	34.6	25.3	12.4
Decreased refined sugar in my diet	N	68	104	109	65	33
	%	17.9	27.4	28.8	17.2	8.7
Eat high-fiber foods	N	44	57	125	103	50
	%	11.6	15.0	33.0	27.2	13.2
Exercise 30 mins 5 days a week	N	64	65	120	78	52
	%	16.9	17.2	31.7	20.6	13.7
Incorporated low-fat foods in diet	N	53	46	123	96	61
	%	14.0	12.1	32.5	25.3	16.1
Periods irregularities						
Use hormonal preparations	N	165	45	73	51	45
	%	43.5	11.9	19.3	13.5	11.9
Use herbal medications	N	96	71	118	59	35
	%	25.3	18.7	31.1	15.6	9.2
Acne						
Use hormonal preparations	N	204	37	63	44	31
	%	53.8	9.8	16.6	11.6	8.2
Topical creams	N	75	51	110	99	44
	%	19.8	13.5	29.0	26.1	11.6
Using Roaccutane pills	N	222	49	61	28	19
	%	58.6	12.9	16.1	7.4	5.0
Infertility						
Using clomiphene	N	196	33	67	31	52
	%	51.7	8.7	17.7	8.2	13.7
Try to reduce weight	N	100	41	104	58	76
	%	26.4	10.8	27.4	15.3	20.1
Laparoscopic ovarian drilling	N	241	42	65	19	12
	%	63.6	11.1	17.2	5.0	3.2

The relationship between knowledge about PCOS and various demographic factors shows that the western region (N=134) is significantly associated with higher knowledge (p<0.001). Participants with monthly income >10,000 SAR (N=123) show significantly higher knowledge levels (p=0.038). Healthcare workers (N=64) and individuals diagnosed with PCOS (N=135) are also associated significantly with high knowledge levels (p=0.003, <0.001). The participants' age, educational level, marital status, nationality, and BMI do not significantly impact knowledge about PCOS. Interestingly, even those without a formal PCOS diagnosis exhibit variable knowledge levels (Table 4).

**Table 4:** Association of knowledge about PCOS with demographic data PCOS: polycystic ovary syndrome.

		Knowledge About PCOS		Significance Value
		Poor Knowledge (N)	High Knowledge (N)	
Age	18-24 years	37	67	0.093
	25-29 years	23	68	
	30-39 years	19	44	
	40-45 years	20	36	
	>46 years	30	35	
Marital status	Married	68	143	0.602
	Single	53	96	
	Widow/divorced	8	11	
Educational level	High school degree	33	45	0.162
	Bachelor's degree	89	184	
	Master's degree	7	21	
Nationality	Non-Saudi	9	14	0.595
	Saudi	120	236	
Residence place	Eastern region	12	62	<0.001
	Northern region	18	31	
	Southern region	7	23	
	Western region	92	134	
Monthly income	<10,000 SAR	42	81	0.038
	>10,000 SAR	60	123	
	<25,000 SAR	25	29	
	>25,000 SAR	2	17	
BMI	>30 kg/m^2^	0	2	0.152
	18-24.9 kg/m^2^	85	141	
	25-29.9 kg/m^2^	44	107	
Healthcare workers	No	113	186	0.003
	Yes	16	64	
Diagnosed with PCOS	Medically diagnosed	43	135	<0.001
	Not diagnosed	73	102	
	Suspected	13	13	

Table 5 shows the correlation between attitudes toward polycystic ovary syndrome (PCOS) and various demographic characteristics. Significantly more positive attitudes are observed among individuals aged 25-29 years (N=72) (p=0.045) residing in the western region (N=146) (p=0.007), healthcare workers (N=64) (p=0.031), and those medically diagnosed with PCOS (N=141) (p<0.001). No significant associations are found for age, marital status, educational level, nationality, monthly income, or BMI categories.

**Table 5:** Association of attitude toward PCOS with demographic data PCOS: polycystic ovary syndrome.

		Attitude Toward PCOS		Significant Value
		Negative Attitude (N)	Positive Attitude (N)	
Age	18-24 years	36	68	0.045
	25-29 years	19	72	
	30-39 years	14	49	
	40-45 years	18	38	
	>46 years	26	39	
Marital status	Married	62	149	0.901
	Single	46	103	
	Widow/divorced	5	14	
Educational level	High school degree	25	53	0.836
	Bachelor's degree	79	194	
	Master's degree	9	19	
Nationality	Non-Saudi	5	18	0.382
	Saudi	108	248	
Residence place	Eastern region	11	63	0.007
	Northern region	12	37	
	Southern region	10	20	
	Western region	80	146	
Monthly income	<10,000 SAR	35	88	0.888
	>10,000 SAR	54	129	
	<25,000 SAR	17	37	
	>25,000 SAR	7	12	
BMI	>30 kg/m^2^	0	2	0.911
	18-24.9 kg/m^2^	69	157	
	25-29.9 kg/m^2^	44	107	
Healthcare workers	No	97	202	0.031
	Yes	16	64	
Diagnosed with PCOS	Medically diagnosed	37	141	<0.001
	Not diagnosed	70	105	
	Suspected	6	20	

Table 6 shows the relationship between lifestyle modification practices for polycystic ovary syndrome (PCOS) and various demographic factors. There are significant associations for age 25-29 years (N=63) (p<0.001), married (N=139) (p<0.001), Bachelor's educational (N=156) (p=0.007), and residence of Kingdom of Saudi Arabia (KSA) (N=203) (p<0.001). Additionally, a significant association is observed for PCOS diagnosis (N=107) (p=0.048), with medically diagnosed individuals being more inclined toward positive practices. However, no significant correlations are found for nationality, monthly income, BMI, or healthcare worker status.

**Table 6:** Association of practice regarding lifestyle modifications in PCOS with different features PCOS: polycystic ovary syndrome.

		Practice Regarding Lifestyle Modifications in PCOS		Significant Value
		Negative Practice (N)	Positive Practice (N)	
Age	18-24 years	64	40	<0.001
	25-29 years	28	63	
	30-39 years	22	41	
	40-45 years	29	27	
	>46 years	23	42	
Marital status	Married	72	139	<0.001
	Single	87	62	
	Widow/divorced	7	12	
Educational level	High school degree	43	35	0.007
	Bachelor's degree	117	156	
	Master's degree	6	22	
Nationality	Non-Saudi	13	10	0.204
	Saudi	153	203	
Residence place	Eastern region	11	63	<0.001
	Northern region	26	23	
	Southern region	16	14	
	Western region	113	113	
Monthly income	<10,000 SAR	46	77	0.259
	>10,000 SAR	86	97	
	<25,000 SAR	27	27	
	>25,000 SAR	7	12	
BMI	>30 kg/m^2^	0	2	0.178
	18-24.9 kg/m^2^	106	120	
	25-29.9 kg/m^2^	60	91	
Healthcare workers	No	126	173	0.208
	Yes	40	40	
Diagnosed with PCOS	Medically diagnosed	71	107	0.048
	Not diagnosed	78	97	
	Suspected	17	9	

## Discussion

PCOS, a common disorder in women, is linked to metabolic problems. Lifestyle changes are crucial for management, but women may have misconceptions and adherence issues. Our study shed light on the knowledge, attitudes, and practices of lifestyle modifications among Saudi women diagnosed with polycystic ovary syndrome (PCOS).

Most participants were young adults, in line with PCOS onset patterns. This aligns with previous findings that PCOS often affects women aged 18-44 years [2,10]. Additionally, a substantial number were married, highlighting the need for early detection and management. In terms of education, 72% of participants had a Bachelor's degree, suggesting that higher education might not necessarily correlate with greater PCOS awareness. This finding contradicts some previous studies that found a positive association between education level and PCOS knowledge [11]. It emphasizes the need for educational initiatives targeting all strata of society. Regarding nationality, the majority of participants were Saudi, reflecting the country's population. However, PCOS's impact can vary among ethnicities due to genetics and environment, necessitating further research [12].

Most participants primarily relied on healthcare professionals for PCOS information, aligning with their vital role in diagnosis and education. However, low use of social media and the internet indicates room for improved public awareness through these sources, potentially leading to earlier PCOS diagnosis and better management [13].

Most participants had a good level of knowledge about PCOS, including its symptoms and potential complications. This finding is in contrast with previous research that demonstrated a generally low level of PCOS knowledge among women diagnosed [14]. It is reassuring that participants recognized the links between PCOS and diabetes, heart diseases, infertility, and mental health issues. Such awareness is vital for comprehensive PCOS management.

The positive attitudes toward PCOS were also notable. Participants recognized various treatment options, including hormonal therapy, anti-diabetic medications, symptomatic treatments, and surgery. Weight reduction was seen as a potential means to improve PCOS symptoms, reflecting the importance of lifestyle modifications in PCOS management. This aligns with previous studies emphasizing the role of lifestyle modifications in improving PCOS outcomes [15].

The majority of participants reported no family history of PCOS. This contradicts the known hereditary component of PCOS, which suggests that there might be underreporting or a lack of awareness within families about PCOS cases. Previous studies show that family history is an independent risk factor for PCOS [16]. Educating families about the condition and its potential genetic link may lead to earlier detection of family members at risk.

The relationship between knowledge about PCOS and various demographic factors, such as age, educational level, marital status, nationality, and BMI, did not significantly impact PCOS knowledge. This finding contrasts with previous research indicating that education level could influence PCOS knowledge [17]. However, participants with PCOS diagnosis exhibited significantly higher knowledge levels, suggesting that awareness campaigns should target both diagnosed and undiagnosed individuals.

Significant associations between attitude toward PCOS and age, region, healthcare worker, and diagnosis status were found. Medically diagnosed participants were more likely to have a positive attitude. This aligns with the concept that diagnosis and awareness of one's condition can lead to more positive attitudes and proactive management [18]. While other demographic factors did not show significant associations with attitude, it is essential to recognize that a positive attitude is a crucial factor in motivating individuals to adhere to lifestyle modifications and treatment regimens [19].

Regarding the association between lifestyle modification practices related to PCOS and various participant characteristics, several significant associations were found, it is worth noting a trend where participants with a medical PCOS diagnosis were more likely to have positive lifestyle modification practices, and this trend is statistically significant [20].

Our study highlights the importance of inclusive educational initiatives to raise awareness about PCOS, particularly addressing the genetic aspect due to low reported family history. Healthcare providers must offer comprehensive information, emphasizing lifestyle modifications. Online resources and support groups can supplement these efforts, fostering a sense of community among PCOS individuals.

This study has limitations, including its relatively small sample size and potential selection bias. Future research could include a larger and more diverse sample to enhance generalizability. Additionally, qualitative research may provide deeper insights into the experiences and challenges faced by Saudi women with PCOS.

## Conclusions

Healthcare professionals play a pivotal role in providing information about PCOS, reflecting the importance of early diagnosis and patient education. The positive attitudes exhibited by the participants, coupled with their recognition of various treatment options, including lifestyle modifications, are promising indicators of comprehensive PCOS management. Weight reduction, in particular, was recognized as a potential means to alleviate PCOS symptoms, reaffirming the significance of lifestyle modifications in the management of this condition. Future research with larger and more diverse samples is needed, as well as qualitative research to delve deeper into the experiences and challenges faced by Saudi women with PCOS.
